# Predictors of One-Year Renal Function Decline in Type 2 Diabetes: Implications for Metabolic Target Management

**DOI:** 10.3390/jcm15020499

**Published:** 2026-01-08

**Authors:** Anudari Batbold, Narangerel Bayarmagnai, Oyumaa Davaasuren, Dorjzodov Dashdorj, Ankhlan Boldbaatar, Azzaya Sodnomjamts, Narkhajid Galsanjigmed, Altaisaikhan Khasag, Oyuntugs Byambasukh

**Affiliations:** 1 Department of Endocrinology, School of Medicine, Mongolian National University of Medical Sciences, Ulaanbaatar 14210, Mongolia; anudari@mnums.edu.mn (A.B.); amd24e306@gt.mnums.edu.mn (O.D.); ankhlan@mnums.edu.mn (A.B.); azzaya.s@mnums.edu.mn (A.S.); narkhajid@mnums.edu.mn (N.G.); altaisaikhan@mnums.edu.mn (A.K.); 2 Department of Internal Medicine, Mongolia Japan Hospital, Mongolian National University of Medical Sciences, Ulaanbaatar 14210, Mongolia; 3 Post Graduate Training Institute, Mongolian National University of Medical Sciences, Ulaanbaatar 14210, Mongolia; pmm17d444@st.mnums.edu.mn (N.B.); pti1230080@pt.mnums.edu.mn (D.D.)

**Keywords:** kidney diseases, chronic, HbA1c, BMI, blood pressure, dyslipidemias

## Abstract

**Background**: Early decline in kidney function is a major complication of type 2 diabetes mellitus (T2DM). The extent to which achievement of glycemic, blood pressure, lipid, and weight targets influences short-term renal trajectories remains insufficiently characterized. **Methods**: We conducted a retrospective analysis of 125 T2DM patients who had baseline and 1-year follow-up eGFR measurements at the Mongolia Japan Hospital of the MNUMS during 2023–2024. Clinical and metabolic control was categorized using standard thresholds. Renal outcomes were assessed using absolute 1-year eGFR change and the occurrence of ≥30% decline. **Results**: Mean eGFR declined from 91.2 ± 24.1 to 88.4 ± 25.5 mL/min/1.73 m^2^ over one year (mean change −3.77 ± 11.3 mL/min/1.73 m^2^); 7.2% experienced ≥30% decline. Individuals with pre-existing CKD showed significantly greater deterioration than those without (interaction *p* < 0.001). Poor glycemic control was consistently associated with greater decline: participants with HbA1c > 7.5% had a significant reduction and regression analyses showed an additional adjusted decline of −4.7 mL/min/1.73 m^2^ (*p* = 0.029) compared with those at target. Elevated blood pressure (>130/80 mmHg) was also associated with greater annual decline (adjusted β = −6.40, *p* = 0.024). Lower BMI (<25 kg/m^2^) predicted larger decreases in eGFR in both CKD and non-CKD groups. Lipid target achievement demonstrated small, inconsistent associations with renal outcomes. **Conclusions**: In this clinical cohort, poor glycemic control and elevated blood pressure were the strongest modifiable predictors of short-term kidney function decline, particularly among individuals without pre-existing CKD.

## 1. Introduction

Chronic kidney disease (CKD) is one of the most serious complications of type 2 diabetes mellitus (T2DM), contributing substantially to end-stage renal disease, cardiovascular morbidity, and premature mortality worldwide [1,2,3]. An estimated one-third of individuals with T2DM develop diabetic kidney disease (DKD) [1], and even modest annual reductions in estimated glomerular filtration rate (eGFR) significantly elevate long-term risks [4,5]. Identifying patients at risk of accelerated decline is therefore critical for timely intervention.

Despite strong guideline recommendations [6,7,8,9], many adults with T2DM do not achieve metabolic or cardiovascular treatment targets [10,11]. Achieving optimal glycemic control, maintaining healthy body weight, managing blood pressure, and controlling dyslipidemia are all recognized as key strategies to slow renal deterioration, yet their relative influence on short-term (1-year) eGFR change remains incompletely understood [10,11,12,13]. Short-term eGFR decline is increasingly recognized as a sensitive predictor of future kidney failure, but evidence is mixed regarding which modifiable factors exert the strongest effects-particularly in real-world clinical settings [1,4,10].

This knowledge gap is especially relevant in countries undergoing rapid epidemiological transitions, such as Mongolia, where the prevalence of diabetes and its complications has risen sharply in recent years [14,15,16,17]. Limited longitudinal data exist describing renal outcomes or the impact of metabolic target achievement in Mongolian adults with T2DM, and real-world evidence is needed to guide early detection and risk stratification in clinical practice.

In this study, we assessed one-year kidney function change among adults with T2DM and evaluated whether achievement of key metabolic targets—glycemic control, blood pressure, BMI, and lipid parameters—was associated with eGFR decline. Because pre-existing CKD strongly influences progression rates, analyses were stratified by CKD status. By integrating repeated-measures models and regression analyses, we aimed to clarify which metabolic domains are most relevant to early renal deterioration and provide clinically applicable insights to support improved management strategies.

## 2. Materials and Methods

### 2.1. Study Participants

This observational study analyzed clinical data from adults with T2DM who received care at the Endocrine Wards of the Mongolia–Japan Hospital at the Mongolian National University of Medical Sciences (MNUMS) between 2023 and 2024. Medical records from 424 individuals were initially screened.

Patients with incomplete key variables—such as HbA1c, BMI, or serum creatinine at either baseline or one-year follow-up—were excluded. Individuals with conditions known to substantially distort renal function trajectories, including active malignancy or end-stage renal disease, were also removed. In addition, four participants classified as underweight (BMI < 18.5 kg/m^2^) were excluded because their small subgroup size limited meaningful analysis. After applying all exclusion criteria, 316 participants were included in the cross-sectional metabolic profile analysis, and 135 individuals with complete baseline and follow-up eGFR measurements formed the longitudinal cohort (Figure S1). Because this was a retrospective study based on available clinical data, a formal a priori sample size calculation was not feasible. However, post-hoc power analyses based on observed mean differences in annual eGFR change across major metabolic categories demonstrated statistical power exceeding 80% for the primary comparisons, supporting the adequacy of the sample size. To ensure that longitudinal changes reflected chronic kidney function trajectories rather than acute kidney injury or measurement error, eGFR values were screened for physiologic plausibility. Extreme annual percent changes (>+50% or <−50%) were excluded as outliers, as such changes are well beyond typical biological and analytic variation in eGFR and inconsistent with established patterns of chronic kidney disease progression [18,19]. Ten individuals met these exclusion criteria (six showing extreme increases and four showing extreme decreases) and were removed. Among the remaining participants, annual percent changes in eGFR ranged from −40.33% to +29.58%, reflecting the observed variation within the analytic cohort, and these data were retained for analysis. After this step, 125 participants constituted the final longitudinal analytic cohort.

The study adhered to the principles of the Declaration of Helsinki and received ethical approval from the Medical Ethics Committee of MNUMS (Approval No. 24/Z-07).

### 2.2. Data Collection and Study Variables

Demographic, clinical, and laboratory variables were extracted from hospital electronic medical records. These included age, sex, BMI, blood pressure, diabetes duration, diabetes treatment modality, and the presence of diabetes-related complications. Laboratory variables comprised HbA1c and lipid parameters (total cholesterol, LDL-C, HDL-C, and triglycerides), using hospital-certified automated analyzers measured using standardized laboratory protocols.

To ensure clinical interpretability and alignment with international guidelines, variables were categorized as follows [6,7,8,9]:

- BMI: <25 kg/m^2^ (normal weight) and ≥25 kg/m^2^ (overweight/obese)
- Blood pressure: <130/80 mmHg (at target) and ≥130/80 mmHg (not at target)
- Lipid targets: LDL-C < 2.6 mmol/L, HDL-C meeting ADA sex-specific thresholds, and triglycerides < 1.7 mmol/L. Lipid target achievement was defined primarily according to LDL-cholesterol levels (<2.6 mmol/L), consistent with international guideline recommendations. HDL-cholesterol and triglyceride levels were analyzed descriptively but were not used to define lipid target status.
- HbA1c categories: <6.5% (well controlled), 6.5–7.5% (moderately controlled), and >7.5% (poorly controlled)

Diabetes-related complications, including neuropathy and retinopathy, were recorded as present or absent based on clinical documentation.

### 2.3. Estimation of eGFR and Definition of Outcomes

Estimated glomerular filtration rate (eGFR) was calculated using the CKD-EPI creatinine-based equation [20]. CKD stage was defined according to KDIGO guidelines based on baseline eGFR values [21]. Baseline eGFR was defined as the first available outpatient serum creatinine measurement during the study period, while the one-year eGFR was defined as the value obtained closest to 12 months after baseline.

Absolute change: The primary renal outcome for all longitudinal analyses was the absolute one-year change (difference between follow-up and baseline eGFR, and percent change was defined as the relative percentage difference over the same interval) in eGFR, calculated as [19]:

eGFR_change_ = eGFR_1yr_ − eGFR_baseline_

eGFR_pct_ = [(eGFR_1yr_ − eGFR_baseline_) / eGFR_baseline_] × 100

Percent change: Percent change was also calculated to facilitate descriptive comparisons. A ≥30% decline in eGFR was additionally examined as a secondary descriptive indicator of rapid progression, consistent with KDIGO guidelines [21]. However, because only nine individuals met this criterion, it was not used as a regression outcome, and analyses instead focused on continuous eGFR change, which provided adequate statistical power.

Baseline CKD status was determined using documented clinical diagnoses in the hospital medical records and baseline eGFR values. Diagnoses had been made by treating physicians in routine clinical care according to KDIGO-based criteria. For research analyses, we did not attempt to diagnose new or incident CKD during follow-up, as albuminuria measurements and repeated long-term eGFR data were not consistently available. KDIGO eGFR categories (G1–G4) were used descriptively to characterize baseline kidney function [21] and to stratify analyses of eGFR trajectories, but not to establish new CKD diagnoses.

### 2.4. Statistical Analysis

Descriptive statistics summarized baseline clinical and metabolic characteristics. Continuous variables were summarized using mean ± standard deviation for approximately normally distributed data and median with range for non-normally distributed variables, and frequencies and percentages for categorical variables. Group differences across HbA1c categories were examined using one-way ANOVA for continuous variables and Pearson’s chi-square tests for categorical variables.

To evaluate renal function trajectories, paired t-tests compared baseline and one-year eGFR within each metabolic subgroup. Repeated-measures General Linear Models (GLM) were applied to estimate marginal means, assess the main effect of time, and evaluate interaction terms (time × metabolic category), stratified by CKD status.

The primary multivariable analyses used linear regression, with absolute one-year eGFR change (mL/min/1.73 m^2^) as the dependent variable. Unadjusted models evaluated each metabolic target independently. Adjusted models included age, sex, diabetes duration, treatment modality, pre-existing CKD, and use of statins and antihypertensive agents. Regression coefficients were reported with 95% confidence intervals.

All statistical analyses were performed using IBM SPSS version 28.0 (IBM Corp., Armonk, NY, USA). A *p*-value of <0.05 was considered statistically significant for all analyses.

## 3. Results

### 3.1. General Characteristics of the Study Population

The study included 125 adults with diabetes, of whom 51 (40.8%) were male. The mean age was 58.1 ± 10.8 years, and the mean BMI was 29.5 ± 4.9 kg/m^2^. Mean systolic and diastolic blood pressures were 128.6 ± 17.2 mmHg and 84.0 ± 11.8 mmHg, respectively. The mean duration of diabetes was 9.7 ± 6.8 years, and 39.2% of participants (n = 49) had at least one diabetes-related complication.

Based on baseline eGFR and KDIGO staging criteria, 83 participants (66.4%) were classified as G1 (eGFR ≥ 90 mL/min/1.73 m^2^), 27 (21.6%) as G2 (eGFR 60–89), 8 (6.4%) as G3a (eGFR 45–59), 6 (4.8%) as G3b (eGFR 30–44), and 1 participant (0.8%) as G4 (eGFR 15–29). Overall, 42 participants (33.6%) had reduced kidney function corresponding to CKD stage G2 or higher at baseline.

The mean HbA1c of the study cohort was 9.0 ± 2.8%. Glycemic control distribution showed that 19 participants (15.2%) had HbA1c < 6.5%, 35 (28.0%) had HbA1c 6.5–7.5%, and 71 (56.8%) had HbA1c ≥ 7.5%. Table 1 summarizes the detailed characteristics stratified by HbA1c category. Sex distribution did not differ significantly across groups (*p* = 0.757). Treatment patterns varied markedly (*p* = 0.006), with insulin or combined insulin–oral regimens used more frequently among individuals with HbA1c ≥ 7.5%. The prevalence of diabetic complications also differed significantly (*p* = 0.007), increasing from 31.6% among those with HbA1c < 6.5% to 46.5% among those with HbA1c ≥ 7.5%. Post-hoc analysis of the chi-square test for treatment distribution revealed that participants with HbA1c < 6.5% were significantly more likely to receive no pharmacologic therapy or lifestyle management alone, whereas those with HbA1c ≥ 7.5% were significantly less likely to be untreated and more frequently received glucose-lowering medications.

### 3.2. Control of Diabetes-Related Comorbidities and Metabolic Targets

Most participants were overweight or obese, with BMI values ranging from 17.2 to 44.0 kg/m^2^. Blood pressure values showed wide variation, with systolic pressures between 90 and 200 mmHg and diastolic pressures between 60 and 140 mmHg. Lipid measurements also exhibited substantial variability: mean triglycerides were 2.32 ± 1.62 mmol/L, mean HDL-C was 2.40 ± 10.10 mmol/L, and mean LDL-C was 3.77 ± 4.51 mmol/L.

**Figure 1:**
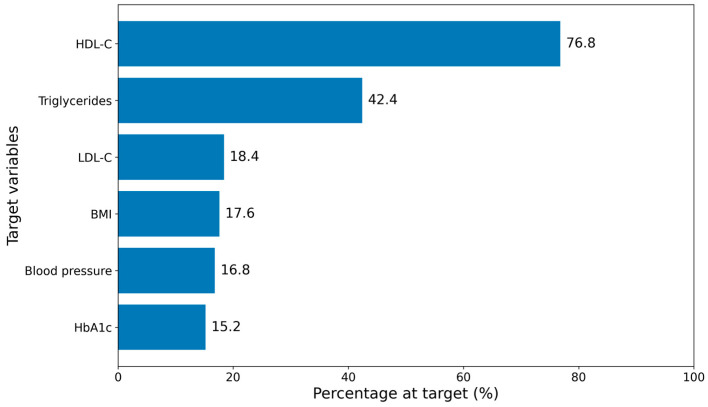
Achievement of metabolic and clinical targets.

Achievement of guideline-recommended metabolic targets was generally low. Only 15.2% of participants attained the HbA1c target, 16.8% achieved the blood pressure goal of <130/80 mmHg, and 17.6% had a normal BMI. Among lipid markers, 20.8% attained LDL-C < 2.6 mmol/L, 76.8% met sex-specific HDL-C criteria, and 45.6% had triglycerides < 1.7 mmol/L (Figure 1).

Medication patterns reflected important treatment gaps. Antihypertensive therapy was indicated in 101 participants (80.8%), yet only 64.0% were receiving treatment. Similarly, statin therapy was indicated for 68 participants (54.4%), but only 47.2% were on a statin.

### 3.3. Renal Function Decline and Its Association with Metabolic Targets

Baseline eGFR ranged widely, from 18.2 to 160.3 mL/min/1.73 m^2^, with a mean of 91.2 ± 24.1 mL/min/1.73 m^2^. After one year, mean eGFR declined to 88.4 ± 25.5 mL/min/1.73 m^2^, corresponding to an average annual reduction of −3.77 ± 11.3 mL/min/1.73 m^2^ (percent change −4.6 ± 14.4%). Nine participants (7.2%) experienced a ≥30% decline. Rapid decline was not associated with sex (*p* = 0.610) or treatment modality (*p* = 0.447). However, pre-existing CKD was strongly associated with rapid decline: 20.8% of those with CKD experienced ≥ 30% loss compared with 4.9% without CKD (*p* = 0.024). Annual eGFR change did not differ significantly across diabetes treatment modalities, including no pharmacologic treatment, lifestyle modification only, oral glucose-lowering agents, insulin therapy, or combination therapy (all *p* > 0.05).

#### 3.3.1. Longitudinal eGFR Change by CKD Status

**Figure 2:**
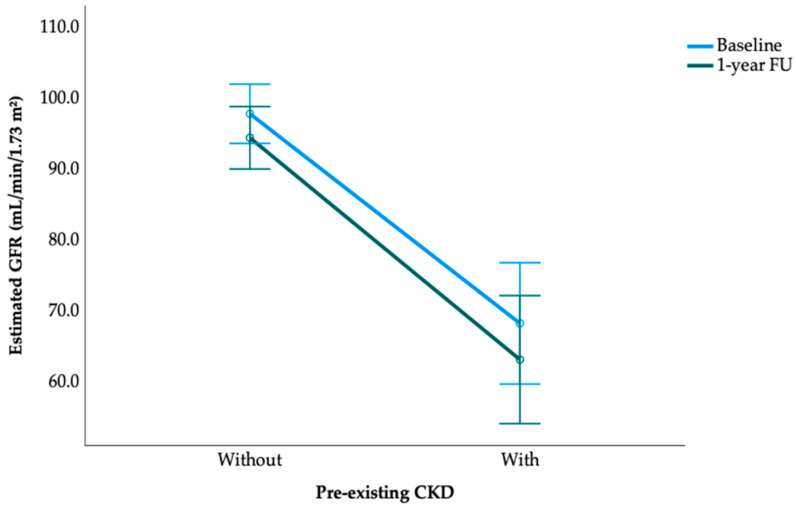
Change in eGFR from baseline to 1-year follow-up among participants with and without pre-existing CKD.

Repeated-measures analysis confirmed a significant deterioration in eGFR over time (F = 108.923, *p* < 0.001). A strong time × CKD interaction (F = 64.234, *p* < 0.001) indicated that participants with pre-existing CKD had a much steeper decline. As shown in Figure 2, those without CKD declined modestly, whereas those with CKD exhibited substantially lower eGFR values at both baseline and follow-up (between-subjects F = 855.889, *p* < 0.001).

#### 3.3.2. Glycemic Control and eGFR Decline

Among individuals without CKD, eGFR changes differed across HbA1c categories (Table 2). Participants with HbA1c < 6.5% showed a small, non-significant increase (+1.96 mL/min/1.73 m^2^; *p* = 0.483), those with HbA1c 6.5–7.5% had a mild decline (−1.65; *p* = 0.444), while those with HbA1c > 7.5% exhibited a substantial and statistically significant reduction of −5.83 mL/min/1.73 m^2^ (*p* < 0.001). In contrast, among participants with CKD, declines were larger in absolute magnitude–especially in the HbA1c > 7.5% group (−6.89; *p* = 0.035)–but did not differ significantly across categories.

#### 3.3.3. BMI and eGFR Decline

BMI demonstrated clinically meaningful patterns. Among non-CKD participants, those with BMI < 25 kg/m^2^ experienced a significant mean decline of −6.84 mL/min/1.73 m^2^ (*p* = 0.010), compared with −2.58 mL/min/1.73 m^2^ (*p* = 0.043) among those with BMI ≥ 25. In the CKD subgroup, declines were larger but not statistically significant: −12.47 mL/min/1.73 m^2^ for BMI < 25 and −3.18 mL/min/1.73 m^2^ for BMI ≥ 25 (Table 2).

#### 3.3.4. Blood Pressure and eGFR Decline

Blood pressure showed consistent directional effects. In the non-CKD group, individuals with BP ≥ 130/80 mmHg experienced a significant mean decline of −4.19 mL/min/1.73 m^2^ (*p* < 0.001), whereas those below this threshold showed no meaningful change (+0.05; *p* = 0.985). In the CKD group, declines were numerically similar (−5.23 vs. −3.85) but did not reach significance (Table 2).

#### 3.3.5. Lipid Targets and eGFR Decline

Lipid target status showed smaller effect sizes. In the non-CKD group, participants not meeting lipid targets declined by −3.43 mL/min/1.73 m^2^ (*p* = 0.004), compared with −2.64 mL/min/1.73 m^2^ (*p* = 0.646) among those at target. Among those with CKD, declines were −7.52 and −5.01 mL/min/1.73 m^2^, respectively, but did not reach significance (Table 2).

#### 3.3.6. Regression Analysis of Metabolic Risk Factors

Regression analyses evaluating predictors of annual eGFR change showed that failing to achieve glycemic targets was associated with a −4.73 mL/min/1.73 m^2^ greater decline (95% CI: −8.94 to −0.52; *p* = 0.028). Elevated blood pressure (BP ≥ 130/80) was also associated with significantly steeper decline (−6.48; *p* = 0.021), while higher BMI (≥25 kg/m^2^) was associated with a relative increase of +6.87 mL/min/1.73 m^2^ (*p* = 0.013) compared with the BMI < 25 group. Lipid target status did not predict change (*p* = 0.987). All associations persisted after adjustment for CKD, age, sex, diabetes duration, treatment, and medication use (Table 3).

## 4. Discussion

In this study of adults with type 2 diabetes, we observed a meaningful decline in renal function over one year and identified several clinical and metabolic factors associated with this change. Achieving metabolic targets is widely recognized as a cornerstone of diabetes care, and international guidelines emphasize strict glycemic, blood pressure, lipid, and weight control to prevent microvascular complications—including chronic kidney disease (CKD) [6,7,8,9,21]. Our findings support this principle, demonstrating that failure to meet specific targets, particularly for glycemia and blood pressure, is associated with measurable short-term deterioration in kidney function. These results reinforce the clinical relevance of target-based management strategies and highlight the need for more systematic interventions to ensure timely achievement of recommended goals.

Consistent with a large body of literature, poor glycemic control was one of the strongest predictors of accelerated renal decline. Participants with HbA1c above the recommended threshold experienced an additional loss of approximately 5–6 mL/min/1.73 m^2^ compared with those at target, even after adjusting for pre-existing CKD. This aligns with the well-established dose–response relationship between hyperglycemia and progressive nephropathy observed in landmark studies such as UKPDS and DCCT/EDIC, which demonstrated that even modest elevations in HbA1c contribute to glomerular hyperfiltration, mesangial expansion, and ultimately eGFR decline [22,23]. Importantly, this relationship was detectable even over a relatively short follow-up period, underscoring the importance of early intervention and sustained glycemic control.

Blood pressure also showed a statistically significant association with renal function loss. Individuals with blood pressure ≥ 130/80 mmHg experienced an additional mean decline of approximately 6.4 mL/min/1.73 m^2^. This is consistent with robust evidence that elevated systolic and diastolic pressures increase intraglomerular pressure, promote endothelial injury, and accelerate nephron loss [10,11,24,25,26,27]. Clinical trials such as ADVANCE and ACCORD-BP have similarly shown that intensive blood pressure management reduces the risk of CKD progression in patients with diabetes [28,29]. Our findings reinforce the critical importance of blood pressure monitoring and treatment intensification, particularly because only a minority of participants achieved the recommended BP target.

Interestingly, BMI showed a significant inverse association with renal decline, with higher BMI associated with a smaller decrease in eGFR. This pattern is consistent with the widely described “BMI paradox,” in which lower BMI—not higher BMI—is associated with poorer renal outcomes in both CKD and non-CKD populations [30,31]. Furthermore, direct measures of body composition such as lean mass or fat mass were not available in this cohort. Therefore, lower BMI may reflect sarcopenia, chronic inflammation, nutritional deficits, or other markers of physiological vulnerability, while modestly elevated BMI may indicate greater metabolic or nutritional reserve [30]. Future studies incorporating body composition assessments are needed to clarify the mechanisms underlying the observed BMI–kidney function relationship.

In contrast, lipid target achievement demonstrated no significant association with eGFR decline in either adjusted or unadjusted models. While dyslipidemia is mechanistically linked with renal microvascular injury through pathways involving oxidative stress and endothelial dysfunction [32], clinical evidence for lipids as short-term predictors of eGFR change remains inconsistent. The absence of a detectable association in this study may reflect the short follow-up duration, the heterogeneity of lipid patterns, or insufficient statistical power.

Compared with reports from other regions—including Europe, East Asia, and North America—the proportion of participants achieving metabolic targets in our cohort was notably lower [33,34,35,36]. Studies have reported HbA1c target achievement rates ranging from 30–60% in high-income settings [9,35,36], whereas only 15.2% of our cohort achieved glycemic goals. Similarly, blood pressure control rates in other populations often exceed 40–50% [35,36,37], yet only 16.8% of participants in our sample were within the ADA-recommended range. These disparities likely reflect differences in healthcare access, medication affordability, patient education, and availability of multidisciplinary diabetes care. The low target achievement rates observed here may help explain the higher prevalence of metabolic complications and underscore the need for strengthened chronic disease management strategies.

This study has several strengths. It provides real-world, clinically grounded evidence from a routine care setting, reflecting the complexity and heterogeneity of diabetes management outside controlled trials. The use of continuous one-year eGFR change as the primary outcome offered enhanced sensitivity for detecting metabolic influences on kidney function. Stratification by CKD status allowed clearer interpretation of differential susceptibility to renal decline. Additionally, the use of repeated-measures modeling and regression analyses provided a robust analytical framework for examining both within-person change and between-group differences. Several limitations should also be considered. Although glycemic targets should be individualized in clinical practice based on age, comorbidities, and overall health status, further stratification into multiple individualized target groups was not feasible due to limited subgroup sizes. HbA1c categories were defined using standardized guideline thresholds to facilitate uniform comparisons across groups. We acknowledge that individualized glycemic targets may be higher in older adults or in patients with advanced CKD or significant comorbidity. Baseline albuminuria measurements were not consistently available in the electronic medical records and could not be incorporated into the analysis, which may have resulted in residual confounding of CKD progression risk. The study relied on a one-year follow-up period, which may be insufficient to capture long-term renal trajectories or the full impact of metabolic factors with slower pathophysiologic effects, such as dyslipidemia. The sample size of the longitudinal cohort was modest, limiting statistical power, particularly in subgroup analyses and in the CKD stratum. The small number of individuals experiencing a ≥30% decline prevented meaningful binary outcome modeling. Residual confounding is possible because unmeasured factors such as medication adherence, diet, body composition, or inflammatory markers were not assessed. The absence of significant differences in eGFR decline across treatment categories may reflect the heterogeneity of therapies within each group and underscores the importance of achieved metabolic control rather than treatment category alone. Detailed data on adherence to structured dietary programs or exercise interventions were not consistently available, and lifestyle modification was documented only as a general treatment category. Direct measures of body composition such as lean mass or fat mass were not available in this cohort. Therefore, BMI may reflect underlying sarcopenia, frailty, or nutritional status rather than adiposity alone. Additionally, eGFR values were based on routine clinical measurements, which may have inherent variability. Although baseline CKD diagnoses were available from clinical records, albuminuria data were not consistently available during follow-up. Therefore, we did not assess incident CKD or apply full KDIGO risk stratification incorporating albuminuria. Finally, although the study setting is clinically representative, findings from a single center may not fully generalize to all healthcare environments.

The results of this Mongolian sample show clearly that achieving metabolic targets is not simply a guideline recommendation but a clinical obligation essential for protecting kidney function in people with type 2 diabetes. In this cohort, suboptimal glycemic and blood pressure control were strongly associated with a greater one-year decline in eGFR, indicating that inadequate target achievement has tangible and rapid physiological consequences. The particularly low rates of glycemic, blood pressure, and lipid target attainment observed among participants highlight an urgent need to strengthen routine diabetes management through earlier treatment intensification, consistent follow-up, improved medication access, and enhanced patient self-management support. Although these challenges are not unique to Mongolia, the findings illustrate how insufficient control of key metabolic parameters can accelerate renal deterioration in real-world clinical settings. Ensuring that patients consistently reach and maintain guideline-recommended targets should therefore be regarded as a fundamental quality standard in diabetes care and a priority area for health-system improvement to reduce the growing burden of CKD.

## 5. Conclusions

In this cohort of adults with type 2 diabetes, failure to achieve recommended glycemic and blood pressure targets was associated with significantly greater one-year declines in kidney function, particularly among those without pre-existing CKD. Although BMI and lipid status showed weaker associations, the overall pattern indicates that multiple metabolic domains contribute—directly or indirectly—to early renal vulnerability. The low rates of target attainment observed in this Mongolian sample highlight important gaps in routine diabetes management and signal the need for more proactive, structured approaches to risk-factor control. Strengthening clinical follow-up, optimizing medication use, and supporting sustained metabolic target achievement may help slow the progression of diabetic kidney disease in similar real-world populations.

## Figures and Tables

**Table 1:** Characteristics of the study population (n = 125) by glycemic control (HbA1c categories).

Findings	HbA1c Category			
	<6.5 (n = 19)	6.5–7.5 (n = 35)	≥7.5 (n = 71)	*p*-Value
Age (years)	57.42 ± 10.34	62.40 ± 9.04	56.07 ± 11.14	0.084
Male, % (n)	13.7% (7/51)	23.5% (12/51)	62.7% (32/51)	0.757
Diabetes duration	7.37 ± 6.26	9.34 ± 8.56	10.25 ± 5.82	0.270
HbA1c	6.05 ± 0.32	6.92 ± 0.31	10.85 ± 2.44	NA
Any diabetic complication, % (n)	31.6 (6)	28.6 (10)	46.5 (33)	0.007
Treatment				0.006
No treatment	50.0 (10)	31.4 (11)	2.0 (2)	
Diet and physical activity	16.7 (3)	2.9 (1)	1.0 (1)	
Oral glycemic agents (OGA)	22.2 (4)	48.6 (17)	72.0 (51)	
Insulin	0 (0)	11.4 (4)	17.0 (12)	
Combination of insulin and OGA	10.0 (2)	5.7 (2)	8.0 (6)	

Data are presented as mean ± standard deviation for continuous variables and percentage (number) for categorical variables. Diabetes duration is reported in years, and HbA1c is reported as percentage (%). *p*-values were derived from one-way ANOVA for continuous variables and Pearson’s chi-square test for categorical variables. Post-hoc comparisons for chi-square analyses were performed using standardized residuals with Bonferroni adjustment to identify subgroup differences.

**Table 2:** Estimated GFR at baseline and 1-year follow-up by glycemic and metabolic targets, stratified by pre-existing CKD.

CKD Status	Category	Time	Mean	95% CI	Δ	*p*-Value *	*p*-Value (Time) **	*p*-Value (Int) ***
No pre-existing CKD	HbA1c < 6.5 (n = 15)	Baseline	94.574	85.384–103.764			0.144	0.033
		1-year FU	96.531	86.105–106.957	+1.957	0.483		
	HbA1c = 6.5–7.5 (n = 31)	Baseline	93.370	86.977–99.762				
		1-year FU	91.722	84.470–98.975	−1.648	0.444		
	HbA1c > 7.5 (n = 55)	Baseline	101.178	96.379–105.977				
		1-year FU	95.351	89.906–100.795	−5.827	<0.001		
Pre-existing CKD	HbA1c < 6.5 (n = 4)	Baseline	66.891	33.814–99.968			0.277	0.602
		1-year FU	65.154	33.002–97.305	−1.737	0.794		
	HbA1c = 6.5–7.5 (n = 4)	Baseline	53.510	20.433–86.587				
		1-year FU	52.085	19.933–84.236	−1.425	0.835		
	HbA1c > 7.5 (n = 16)	Baseline	72.216	55.678–88.755				
		1-year	65.330	49.254–81.405	−6.886	0.035		
No pre-existing CKD	BMI < 25 (n = 16)	Baseline	102.742	94.298–111.186			0.003	0.165
		1-year FU	95.906	86.088–105.724	−6.836	0.010		
	BMI > 25 (n = 44)	Baseline	96.116	92.431–99.801				
		1-year FU	93.540	89.255–97.825	−2.576	0.043		
Pre-existing CKD	BMI < 25 (n = 5)	Baseline	68.863	39.285–98.442			0.012	0.118
		1-year FU	56.396	28.170–84.622	−12.467	0.161		
	BMI > 25 (n = 19)	Baseline	68.039	52.866–83.213				
		1-year FU	64.855	50.375–79.335	−3.184	0.180		
No pre-existing CKD	BP < 130/80 (n = 19)	Baseline	101.308	93.044–109.572			0.149	0.139
		1-year FU	101.359	92.233–110.484	+0.051	0.985		
	BP > 130/80 (n = 82)	Baseline	96.988	93.010–100.966				
		1-year FU	92.803	88.410–97.195	−4.185	<0.001		
Pre-existing CKD	BP < 130/80 (n = 2)	Baseline	54.335	8.005–100.664			0.317	0.878
		1-year FU	50.485	5.925–95.044	−3.850	0.485		
	BP > 130/80 (n = 2)	Baseline	69.473	55.504–83.441				
		1-year FU	64.239	50.804–77.674	−5.234	0.058		
No pre-existing CKD	Lipids at target (n = 11)	Baseline	94.018	77.856–110.181			0.244	0.880
		1-year FU	91.377	73.348–109.406	−2.641	0.646		
	Lipids not at target (n = 90)	Baseline	97.998	94.309–101.686				
		1-year FU	94.570	90.456–98.685	−3.428	0.004		
Pre-existing CKD	Lipids at target (n = 2)	Baseline	62.478	−3.614–128.571			0.318	0.840
		1-year FU	54.954	−8.492–118.400	−7.524	0.058		
	Lipids not at target (n = 22)	Baseline	68.460	54.679–82.242				
		1-year FU	63.447	50.217–76.676	−5.013	0.061		

Data are presented as estimated marginal means with 95% confidence intervals derived from repeated-measures general linear models. Δ indicates the mean change in eGFR from baseline to 1-year follow-up. * *p*-values correspond to within-category paired comparisons of baseline versus follow-up eGFR. ** *p*-values indicate the main effect of time within each CKD stratum. *** *p*-values represent the interaction between time and the corresponding metabolic category, indicating whether eGFR trajectories differed between categories.

**Table 3:** Association between metabolic targets and annual eGFR change.

Glycemic and Metabolic Targets	Category	Beta Coefficient	95% CI	*p*-Value
Unadjusted				
Glycemic control	HbA1c < 6.5	0 (Reference)	–	–
	HbA1c > 6.5	−4.731	−8.942 to −0.521	0.028
Blood pressure	BP < 130/80	0 (Reference)	–	–
	BP > 130/80	−6.484	−11.986 to −0.983	0.021
Body weight	BMI < 25	0 (Reference)	–	–
	BMI > 25	+6.869	+1.479 to +12.259	0.013
Lipids	At target	0 (Reference)	–	–
	Not at target	−0.075	−9.323 to +9.172	0.987
Adjusted for pre-existing CKD				
Glycemic control	HbA1c < 6.5	0 (Reference)	–	–
	HbA1c > 6.5	−4.709	−8.941 to −0.478	0.029
Blood pressure	BP < 130/80	0 (Reference)	–	–
	BP > 130/80	−6.401	−11.963 to −0.838	0.024
Body weight	BMI < 25	0 (Reference)	–	–
	BMI > 25	+6.814	+1.385 to +12.242	0.014
Lipids	At target	0 (Reference)	–	–
	Not at target	−0.069	−9.354 to +9.216	0.988

The dependent variable in all models is the annual change in eGFR (mL/min/1.73 m^2^). For each metabolic domain, the clinically recommended target group serves as the reference category. Categorical predictors were included in linear regression analyses using dummy variable coding, allowing estimation of associations with the continuous outcome of annual eGFR change. Adjusted for pre-existing CKD, age, gender, diabetic duration and treatment and statin and anti-hypertensive drug use. Statistical significance was defined as *p* < 0.05.

## Data Availability

The data used to support the findings of this study are available from the corresponding author upon request.

## Supplementary Materials

The following supporting information can be downloaded at: https://www.mdpi.com/article/10.3390/jcm15020499/s1. Figure S1. Flow diagram of participant selection and exclusion.
