# Supplementary figures and images for: Predictors of One-Year Renal Function Decline in Type 2 Diabetes: Implications for Metabolic Target Management
Source: J Clin Med. 2026 Jan 8;15(2):499. doi: 10.3390/jcm15020499 (PMC12841840; doi:10.3390/jcm15020499)

Supplementary Figure

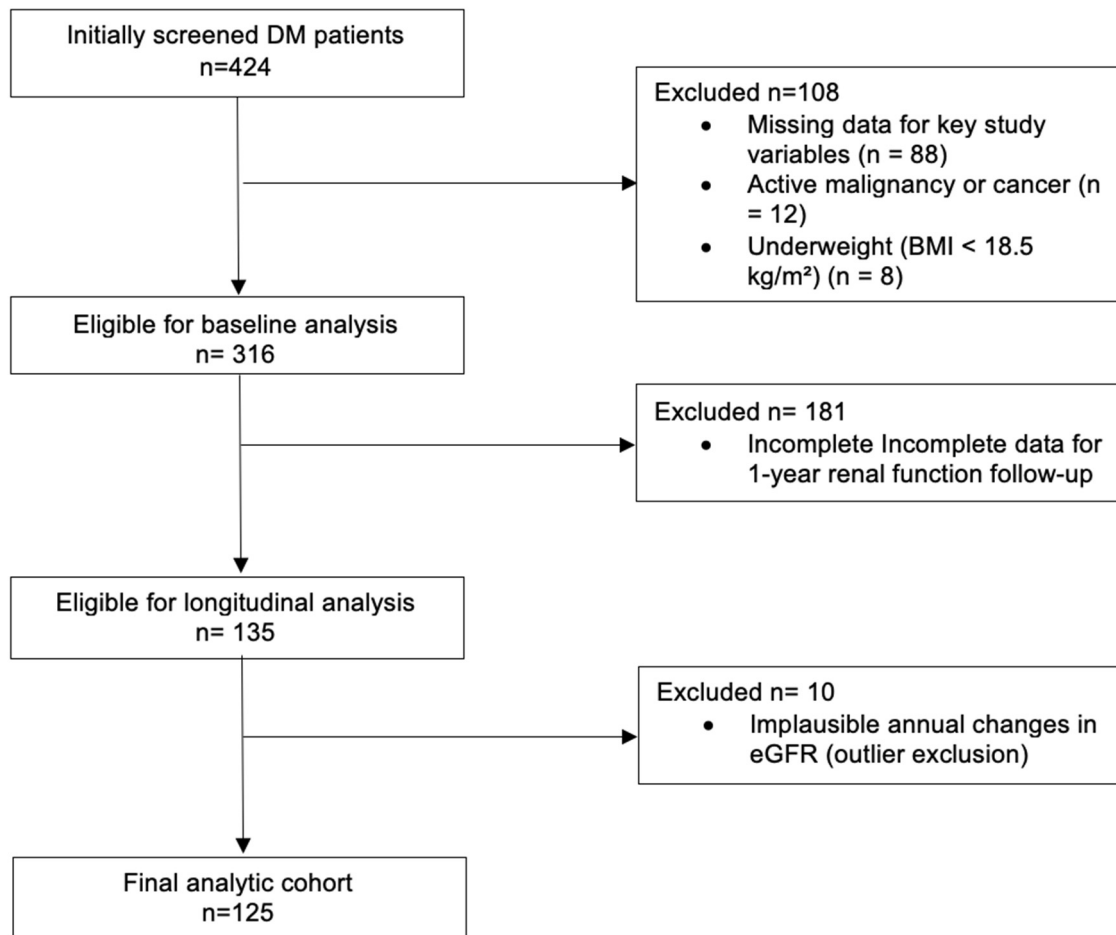

Figure S1. Flow diagram of participant selection and exclusion.

Supplement: Supplementary file 1 [file jcm-15-00499-s001.zip › jcm-4047277-supplementary.pdf]
